# The SEE-IT Trial: emergency medical services Streaming Enabled Evaluation In Trauma: a feasibility randomised controlled trial

**DOI:** 10.1186/s13049-024-01179-0

**Published:** 2024-01-26

**Authors:** Cath Taylor, Lucie Ollis, Richard M. Lyon, Julia Williams, Simon S. Skene, Kate Bennett, Matthew Glover, Scott Munro, Craig Mortimer, the SEE-IT Trial Group, Jill Maben, Carin Magnusson, Heather Gage, Mark Cropley, Janet Holah

**Affiliations:** 1 School of Health Sciences, University of Surrey, Guildford, Surrey UK; 2 Kent, Surrey and Sussex Air Ambulance, Redhill, UK; 3 South East Coast Ambulance Service NHS Foundation Trust, Crawley, West Sussex UK; 4 School of Health and Social Work, University of Hertfordshire, Hatfield, UK; 5 Surrey Clinical Trials Unit, University of Surrey, Guildford, UK; 6 Surrey Health Economics Centre, School of Biosciences, University of Surrey, Guildford, UK; 7 School of Psychological Sciences, University of Surrey, Guildford, UK; 8 Patient Public Involvement and Engagement Lead, Guildford, UK

**Keywords:** Emergency medical services, Emergency medical dispatch, Helicopter emergency medical services, Emergency medical resource, Air ambulance, Pre-hospital, Critical care, Trauma, Smartphone, Video

## Abstract

**Background:**

Use of bystander video livestreaming from scene to Emergency Medical Services (EMS) is becoming increasingly common to aid decision making about the resources required. Possible benefits include earlier, more appropriate dispatch and clinical and financial gains, but evidence is sparse.

**Methods:**

A feasibility randomised controlled trial with an embedded process evaluation and exploratory economic evaluation where working shifts during six trial weeks were randomised 1:1 to use video livestreaming during eligible trauma incidents (using GoodSAM Instant-On-Scene) or standard care only. Pre-defined progression criteria were: (1) ≥ 70% callers (bystanders) with smartphones agreeing and able to activate live stream; (2) ≥ 50% requests to activate resulting in footage being viewed; (3) Helicopter Emergency Medical Services (HEMS) stand-down rate reducing by ≥ 10% as a result of live footage; (4) no evidence of psychological harm in callers or staff/dispatchers. Observational sub-studies included (i) an inner-city EMS who routinely use video livestreaming to explore acceptability in a diverse population; and (ii) staff wellbeing in an EMS not using video livestreaming for comparison to the trial site.

**Results:**

Sixty-two shifts were randomised, including 240 incidents (132 control; 108 intervention). Livestreaming was successful in 53 incidents in the intervention arm. Patient recruitment (to determine appropriateness of dispatch), and caller recruitment (to measure potential harm) were low (58/269, 22% of patients; 4/244, 2% of callers). Two progression criteria were met: (1) 86% of callers with smartphones agreed and were able to activate livestreaming; (2) 85% of requests to activate livestreaming resulted in footage being obtained; and two were indeterminate due to insufficient data: (3) 2/6 (33%) HEMS stand down due to livestreaming; (4) no evidence of psychological harm from survey, observations or interviews, but insufficient survey data from callers or comparison EMS site to be confident. Language barriers and older age were reported in interviews as potential challenges to video livestreaming by dispatchers in the inner-city EMS.

**Conclusions:**

Progression to a definitive RCT is supported by these findings. Bystander video livestreaming from scene is feasible to implement, acceptable to both 999 callers and dispatchers, and may aid dispatch decision-making. Further assessment of unintended consequences, benefits and harm is required.

*Trial registration*. ISRCTN 11449333 (22 March 2022). https://www.isrctn.com/ISRCTN11449333

**Supplementary Information:**

The online version contains supplementary material available at 10.1186/s13049-024-01179-0.

## Introduction

### Background and rationale

Accurate and timely response of emergency medical services (EMS) after trauma incidents is critical to ensure optimal patient outcomes and prevent serious injury or death [1]. Currently, most ambulance services in the UK rely on lay public callers to relay accurate information verbally via the telephone about the state of the patient(s) and what has happened at the scene. Due to limited medical knowledge and/or training, the emotional impact of witnessing an incident, language barriers, and subjectivity, lay public callers often do not provide accurate information to the emergency operations centre (EOC) [2, 3]. Misinformation can lead to either under-resourcing or over-resourcing of EMS [4–7], which can make it challenging to initiate timely and accurate dispatch of EMS and can mean essential critical care resources may not be available to those who need them most.

The use of video livestreaming from bystanders at scenes of medical incidents is becoming increasingly common in the UK and worldwide [8]. Using such technology means that those making decisions about the resources and support required can see the scene and patient(s) involved in incidents. There are potential clinical and financial benefits of improving accuracy and speed of EMS dispatch, but evidence is currently limited. Studies have predominantly focused on out-of-hospital cardiac arrest, with evidence of clinical benefit in this setting [9, 10]. Most of these studies have been simulation-based [11–13] with few studies in real-life settings. Evidence suggests that use of video livestreaming from the scene may impact on decision-making [8, 14] and enable faster and more accurate decisions about EMS resources to be tasked [3]. To our knowledge, two real-life studies have focussed on the impact of video livestreaming from callers [8, 15]. In one study [8], 97% of surveyed callers (108/111) felt that video livestreaming should be implemented into practice. The introduction of a new technology (such as GoodSAM Instant-On-Scene [16]) into the EMS dispatch process is not necessarily straightforward. In addition to the operational impact of introducing a new technology into a busy, emergency control room environment, it is likely to require additional training, and changes to pre-existing protocols and/or dispatch criteria. In addition, the risk of potential harm to members of the public and EMS staff viewing trauma via video livestreaming has not been explored in previous research studies, demonstrating further need for this study.

### Objectives

The aim of this feasibility randomised controlled trial (RCT) was to assess the feasibility of implementing and evaluating GoodSAM Instant-On-Scene [16] (video livestreaming) in a definitive RCT. The main objective was to judge whether a definitive RCT was potentially viable by determining answers to pre-determined progression criteria (see below), and secondary related objectives included::

(i) To obtain data required to inform the design of a definitive RCT.
(ii) To test trial processes including randomisation and data collection methods.
(iii) To embed a process evaluation to test the acceptability and feasibility of using video livestreaming from provider (EMS staff) and public (callers) perspectives.

## Methods

### Trial design

A feasibility RCT with an embedded process evaluation, exploratory economic evaluation and two observational sub-studies. These comprised (i) an inner-city sub-study in an ambulance service already routinely using video livestreaming to explore the acceptability and feasibility of using video livestreaming in a more diverse population; and (ii) a staff wellbeing sub-study in an ambulance service not using video livestreaming to provide comparison to the trial staff. The full trial protocol was published once no further protocol changes were required [17]. Previous versions of the trial protocol can be found on the NIHR website, including a log of amendments [18]. Notable changes after trial commencement included: (i) refinement of incident-level inclusion and exclusion criteria after feasibility testing in the first trial week (refinement of criteria in order to operationalise the protocol; participant inclusion/exclusion remained the same as below); (ii) addition of telephone consent for patients to improve recruitment uptake; (iii) approval for the Research Paramedics to record patient’s names to assist the hospitals with locating and consenting patients; and (iv) addition of a reminder text inviting 999 callers to participate in the survey to improve recruitment uptake.

### Participants

#### Eligibility criteria

Participants included trauma patients, emergency telephone line (999) callers (hereafter “callers”) and EOC staff. EOC staff included Helicopter Emergency Medical Services (HEMS), critical care dispatch staff, together with five Research Paramedics who observed and collected data in all trial shifts (see Box 1 Glossary). The participant inclusion and exclusion criteria can be found in Tables 1 and 2 respectively.

**Table 1:** Participant inclusion criteria (published in protocol paper [17])

Participant type	Main feasibility trial	Inner-city sub-study	Staff wellbeing sub-study
Lay public callers	999 callers during the six trial weeks where the incident involved major trauma (defined as per below)	All 999 callers during observed shifts that involved trauma and were screened by HEMS dispatchers or Advanced Paramedic Practitioners in Critical Cares (APP-CCs) who attempted to use GoodSAM during the call	N/A
EOC staff	All CCPs, HEMS dispatchers, and Research Paramedics	All HEMS dispatchers and APP-CCs	All CCPs and HEMS dispatchers
Trauma patients	All trauma patients during the six trial observation weeks who were the subject of 999 calls involving major trauma, judged by the HEMS dispatcher and/or CCPs as likely to require enhanced dispatch	All trauma patients during observed shifts that involved trauma and were screened by HEMS dispatchers or APP-CCs who attempted to use GoodSAM during the call	N/A

**Table 2:** Participant exclusion criteria (published in protocol paper [17])

Participant type	Main feasibility trial	Inner-city observational sub-study	Staff wellbeing sub-study
Lay public 999 callers	Calls were excluded where: (i) 999 caller was not at the scene; (ii) 999 call originated from a landline; (iii) 999 call originated from another emergency service e.g. police or fire; (iv) 999 calls where resource (excluding community first responder) would arrive on scene before video livestreaming could be activated; (v) 999 call ended before transfer for activation of video livestreaming; (vi) 999 calls where another incident took priority; and (vii) calls where clinical acuity was found to be lower than the threshold for the study (not major trauma) All callers identified by the dispatchers as a child caller (under 16 years old) and those who selected they are under 16 on the 999-caller survey were excluded	All callers identified by the HEMS dispatchers/APP-CCs as a child caller (under 16 years old) and those who selected they are under 16 on the 999-caller survey were excluded	N/A
EOC staff	EOC staff not mentioned in the inclusion criteria	EOC staff not mentioned in the inclusion criteria	EOC staff not mentioned in the inclusion criteria
Trauma patients	Any emergencies of a suspected medical origin (e.g., heart attack or stroke)	Any emergencies of a suspected medical origin (e.g., heart attack or stroke)	N/A

#### Settings and locations where the data were collected

The main feasibility trial was conducted in South-East Coast Ambulance Service NHS Foundation Trust (SECAmb) between its two Emergency Operations Centres (EOCs). The EOCs are responsible for answering emergency calls and dispatching all EMS resources. SECAmb covers 9324 square kilometres across a diverse geographical area including urban, rural, and stretches of motorway. Emergency response for SECAmb also includes access to the Air Ambulance Charity Kent, Surrey, Sussex (KSS, a Helicopter Emergency Medical Service, HEMS). KSS covers the same geographical area, with a population of up to 8 million people.

**Fig. 1:**
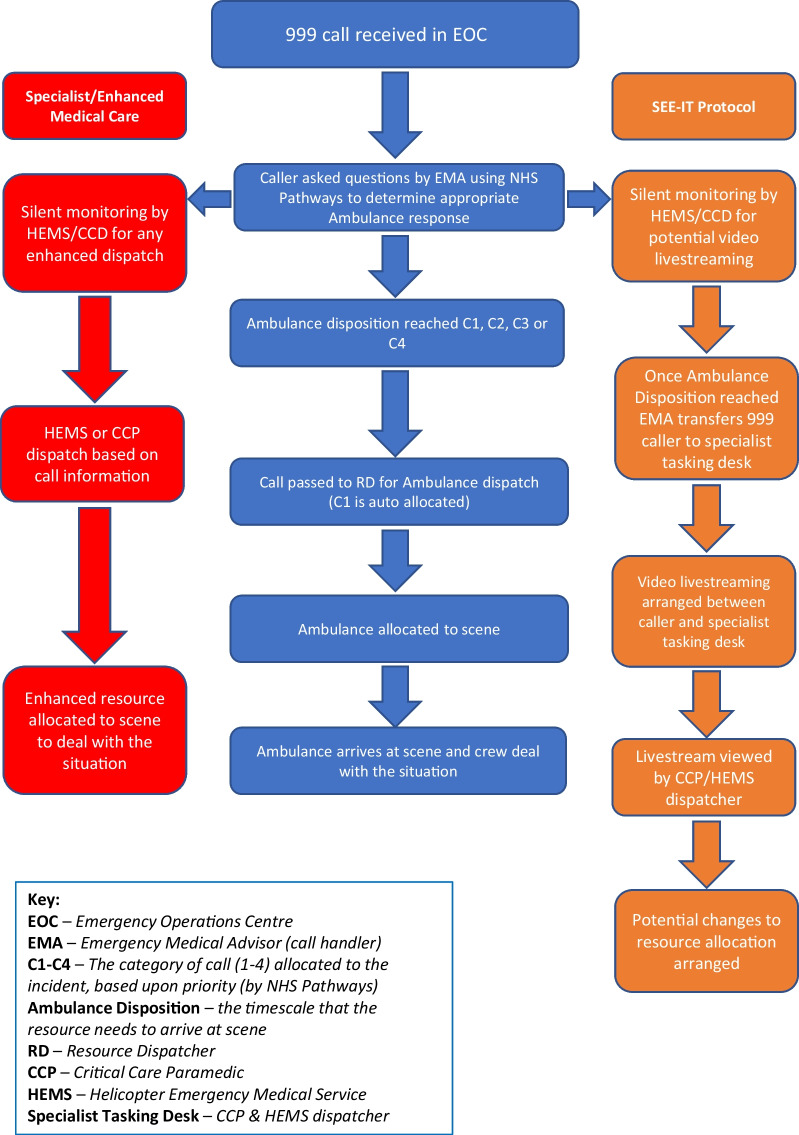
Flowchart of process

A full list of sites is provided in the trial protocol [17]. The observational inner-city sub-study was conducted in London Ambulance Service NHS Trust (LAS, including London’s Air Ambulance Charity, LAA), and the staff wellbeing sub-study was in East of England NHS Trust (EEAST). Figure 1 provides a flow diagram from emergency call to show the operational process at the study site.

#### How participants were identified and consented

Patients and callers were identified through their involvement in an eligible incident during a trial observation shift. Staff were identified through their role as either a HEMS dispatcher or a Critical Care Paramedic (CCP) working on the Critical Care Desk (CCD). Following approval from CAG, written consent was not required for participation of patients or callers during incidents, and for basic information to be retained (estimated sex, age and type of incident/injuries, and resources sent to the scene). Verbal consent was obtained from callers prior to use of video livestreaming, and from patients where possible. Patients (or their guardians/consultees) were approached by research staff in the hospital for consent to access their medical records for up to 3 months post-incident. These data were required to assess the appropriateness of the resource sent to the scene. Callers were sent a text message after the incident to ask for their permission to be sent a survey in 6–8 weeks’ time to assess their wellbeing. The invitation text was sent within 8 h of their call. Staff were sent invitations to participate in the survey via their managers and were asked to indicate willingness to participate in interviews within the survey.

### Interventions

The use of video livestreaming via GoodSAM Instant-on-Scene [16] was tested. An SMS text message was sent to eligible callers via the GoodSAM web platform. Callers were asked to switch to loudspeaker mode, click on the link within the SMS and confirm permission for use of their phone’s camera and microphone. No live streamed video footage was recorded (either on the caller’s phone or in the EOC).

### Procedure

The procedure for calls is illustrated in Fig. 1. Two dispatching systems are in current use in the UK: The Medical Priority Dispatch System (MPDS) and the NHS Pathways systems These are complex triage tools used in the assessment and categorisation of 999 calls. In the trial study site, NHS Pathways is used [19]. In both arms, eligible emergency calls (999 calls in the UK) were initially taken by the Emergency Medical Advisor (EMA, call handler) who followed the standard NHS Pathways triage tool. Concurrently, HEMS dispatchers and the CCD monitored the calls by reading (and/or listening to) information entered by call handlers in to the Computer-aided-dispatch (CAD) system. A study-specific code was entered by the HEMS dispatcher/CCD into the CAD when an incident was thought to meet eligibility criteria. In control arm shifts, when the call ended, standard protocols for dispatch were followed. In intervention arm shifts, the EMA asked callers to stay on the phone allowing transfer to the specialist tasking desk (either the HEMS dispatcher or CCD). Initial dispatch of EMS resources took place during the NHS Pathways call for both arms of the trial as per standard practice. If the caller was successfully transferred, the HEMS dispatcher/CCD confirmed (using a pre-defined script) whether they were using a smartphone and asked if they were willing and able to safely live stream from the scene. Either the HEMS dispatcher or CCD could instigate the video livestreaming, but both could view the obtained footage. Once the HEMS dispatcher/CCD felt they had gathered enough information, video livestreaming was ended, and the caller was thanked for their help.

### Outcomes

The primary outcome of this feasibility RCT was the decision regarding the feasibility of conducting a definitive RCT. This was based on meeting a set of predefined progression criteria (see Table 3), together with consideration of qualitative data (e.g., interviews, observations, and free text questions in surveys). Findings were also reviewed and endorsed by an independent study Steering Committee.

**Table 3:** Progression criteria (published in protocol paper [17])

GREEN; proceed to definitive study—GO	AMBER; consider protocol amendments to improve criteria	RED; do not proceed to main trial—STOP
≥ 70% of callers with smartphones agreeing and able to activate video livestreaming	≥ 50% of callers with smartphones agreeing and able to activate video livestreaming	< 50% of callers with smartphones agreeing and able to activate video livestreaming
≥ 50% of requests to activate video livestreaming resulting in footage being viewed	≥ 30% but < 50% of requests to activate video livestreaming resulting in footage being viewed	< 30% of requests to activate video livestreaming resulting in footage being viewed
Air Ambulance (HEMS) stand-down rate reducing by ≥ 10% and/or change in dispatch decision as a result of live streamed footage confirmed as being appropriate in ≥ 10% cases	Air Ambulance stand-down rate reducing by ≥ 5% and/or change in dispatch decision as a result of live streamed footage confirmed as being appropriate in ≥ 5% cases	No change in Air Ambulance stand-down rate and/or change in dispatch decision as a result of live streamed footage
Rates of psychological harm (based on the survey measures) not significantly greater in 999 callers using video livestreaming compared to those not; and no significant difference in change to psychological harm over time in staff (CCPs, HEMS dispatchers, Research Paramedics) compared to change in staff in a comparison EOC not using video livestreaming/streamed from scene footage	–	Evidence of significantly greater harm in either 999 callers or EOC staff using video livestreaming compared to EOC not using video livestreaming

Secondary outcomes included: (i) Speed of appropriate emergency services dispatch. Speed was measured from initiation time of the 999 call to mobile time of appropriate resource(s) (using time-stamped data from the CAD). ‘Appropriateness’ was based on a set of pre-defined criteria developed through expert consensus from pre-hospital Emergency Medicine experts and used medical records data for up to 3 months post-incident (nature of injuries and treatments received) for all patients that provided consent for this. (ii) Stand-down rate (de-escalation) of enhanced resources (CCP/HEMS); (iii) missed jobs (these were identified by reports run by SECAmb Business Intelligence). This included calls where HEMS or CCP(s) were required at the scene of trauma incidents within trial observation shifts, but where the code to indicate eligibility of the incident for the trial had not been entered into the CAD by the HEMS dispatcher/CCD; (iv) request for further ambulance resources from scene: requests for enhanced dispatch resources (CCP and/or HEMS) from the scene were recorded by the Research Paramedics observing all trial shifts. The outcomes and their data collection and analysis are described in the trial protocol [17].

### Sample size

Based on data from KSS HEMS of six calls per day, we estimated the event rate to be 250 trauma incidents over the six trial weeks (125 allocated to intervention and 125 to control) including approximately 300 patients. This would allow sufficient data to assess the feasibility objectives, providing an estimate of true event rate within a precision ± 0.75 events per day and allowing for the estimation of speed to appropriate dispatch with a standard error of < 5%.

### Randomisation

Six trial weeks took place for one week each month between June and November 2022, comprising up to 14 × 12-h working shifts each week. Each shift was randomised 1:1 to either the intervention (use of GoodSAM) or control condition (standard care) by a statistician at Surrey Clinician Trials Unit. We chose to randomise by shift as a pragmatic option to allow a clear, uniform and decisive means of deciding whether to use GoodSAM or not and to allow us to assess the strengths and limitations of using this method.

A minimisation algorithm was used to ensure balance between day shifts (06:00–18:00) versus night shifts (18:00–06:00) and weekdays (Mon-Thurs) versus weekend days (Fri-Sun). Shifts were only randomised if the HEMS desk and CCD were co-located. In the final trial week, three shifts were used to test the feasibility of randomising by individual call, using a pre-prepared randomisation list. Once the code was entered into the CAD to identify the call as being eligible, the study Research Paramedic opened a sealed envelope and announced to the HEMS dispatcher/CCD if the incident was allocated to the control or intervention condition.

### Blinding

Whilst the use of GoodSAM could not be concealed, the allocation of shifts was concealed until the start of each trial week, at which time posters were displayed around the EOC (visible to all dispatchers and EMAs) detailing the randomisation for that week. Additionally, the Expert Panel were blinded to the assignment of intervention when applying the appropriateness of dispatch criteria.

### Analytical methods

A statistical analysis plan was produced and approved by the independent study Steering Committee prior to undertaking analysis. Analysis focused on providing estimates and confidence intervals to inform the design of a subsequent RCT. The primary analysis was undertaken on an intention-to-treat (ITT) basis, that is, every incident is included in the group to which it was randomised, regardless of the adherence to the randomised intervention, although deviations are noted. The analysis was planned in accordance with the relevant CONSORT guideline extensions for cluster randomised trials, randomised pilot trials and feasibility trials [20, 21]. Data regarding personnel and services dispatched (descriptives and time spent attending) were also used to inform health economic analysis for a future trial.

For measurement of potential harm in staff, surveys were sent pre-trial and post-trial (and at the same time to similar staff in a comparison Ambulance service who were not using livestreaming). Mean scores for the General Health Questionnaire (GHQ-12) [22] and the Impact of Events Scale – Revised (IES-R) [23] were compared using t-tests.

An exploratory health economic analysis was conducted, to inform the design of a potential future economic evaluation and assess the feasibility of collecting resource use, cost, and consequence data related to the dispatch process. Resource use comprised personnel and services dispatched, attending the scene and conveyance. A variety of sources were used to compute unit costs (2021/22 prices) per minute (Additional file 2: Table 1), which were applied to incident ambulance and HEMS resources [24–27]. Intervention costs included service fee for use, time of HEMS dispatchers and CCP review. Exploratory analysis (ITT) was conducted using a cost-consequence framework. Differences in mean ambulance/HEMS costs per incident were estimated and consequences were characterised as the proportion of appropriate dispatch decisions.

### Embedded process evaluation

A mixed methods process evaluation was designed to assess the acceptability and feasibility of implementation of the intervention (video livestreaming) and of the study protocol and processes. Methods included non-participant observation in the main trial site EOC (SECAmb, control and intervention shifts), surveys and semi-structured interviews (with both callers and EOC staff). The surveys were designed predominantly to assess risk of psychological harm (see above for analysis of these) but the caller survey was also designed to collect data on the acceptability of video livestreaming.

The inner-city observational sub-study included non-participant observation of the use of livestreaming by HEMS dispatchers (paramedics) and APP-CCs, a survey sent to callers who were observed using video livestreaming (and invitation to survey respondents to participate in a follow-up semi-structured interview), and semi-structured interviews with EOC staff who used (and/or had access to use) livestreaming for major trauma incidents. The survey and interview questions were designed predominantly to investigate the acceptability of livestreaming and any influences on this (e.g., ethnicity, culture, language). EOC observations were conducted with two prehospital critical care teams that routinely use video livestreaming for trauma—the LAS APP-CCs and LAA HEMS dispatchers.

Observational and interview data were analysed using the Framework Method [28]. Data collection and analysis were underpinned by a number of relevant theoretical frameworks including decision making [29], situational awareness [30], implementation of technology [31–33] and implementation science (Consolidated Framework for Implementation Research, [30]).

## Results

### Recruitment

A total of 62 shifts were randomised: 31 shifts to the control arm and 31 to the intervention arm (see Additional file 1: Fig. S1—consort flowchart). Eight of the night shifts (4 control and 4 intervention) ended at 22:00 due to the CCD not being co-located with the HEMS dispatchers in the East EOC. The shifts included a total of 240 eligible incidents (132 control and 108 intervention). A further 3 shifts were randomised by individual call (a method reported as acceptable and feasible by HEMS dispatchers, CCPs and Research Paramedics). These shifts included 4 eligible calls (2 allocated to intervention and 2 to control), resulting in a total of 244 incidents involving 269 patients (see Additional file 1: Figs. S1 and S2).

The first participant was a staff member recruited on 17 June 2022 by completing the pre-trial staff survey (completed between 17 June 2022 and 30 July 2022). Staff completed the post-trial follow-up survey between 13 December 2022 and 23 January 2023. Patients were recruited from 28 June 2022 until 28 February 2023, up to 3 months after the last trial day (28 November 2022). The recruitment period for callers was from the first trial day (28 June 2022) to the final trial day (28 November 2022). The trial ended at the end of the six planned trial weeks.

### Outcomes and estimation

Numbers analysed, and outcomes in relation to the progression criteria are provided in Table 4. Two of the four progression criteria were confirmed as ‘Green’ (proceed to definitive study) and two were indeterminate. Once calls were transferred successfully (79/110, 72%) and the caller could thereby be asked if they were using a smartphone (72/79, 91%), most with smartphones were able and willing to activate video livestreaming (Criteria 1: 86%). Of these, most requests to activate video livestreaming resulted in footage (Criteria 2: 85%).

**Table 4:** Progression criteria findings

Progression criteria			RAG rating
1: Proportion of callers with smartphones agreeing to and able to activate video livestreaming	n/N (%)	62/72 (86)	Green
	95% CI	(76 to 93)	
2. Number (proportion) of requests to activate that result in GoodSAM footage being viewed by HEMS and/or CCP	n/N (%)	53/62 (85)	Green
*NB: The number of requests to activate is taken as the number of GoodSAM texts received (n* = *62)*	(95% CI)	(74 to 93)	
3. Air Ambulance standdown rate/change in dispatch decision as result of livestreaming	n/N (%)	2/20 (10) ITT*	Indeterminate due to insufficient data
		2/6 (33) PP	
4a. Rates of psychological harm not significantly greater in 999 callers using livestreaming vs. not	Only 4/244 (1.6%) callers completed surveys		
	No evidence of harm from interviews or observations		
4b. No significant difference in change over time in psychological harm in staff within the trial site compared to the comparison site			
Trial site: Pre-intervention IES-R (n = 41)	3.6 (6.2)		
Post-intervention IES-R (n = 25)	2.8 (4.4)		
Change (mean, SD), difference between time points (95% CI)	− 1 (8.5), − 1.0( − 4.5,2.5)		
Pre-intervention GHQ-12 (n = 40)	9.6 (3.4)		
Post-intervention GHQ-12 (n = 25)	9.0 (2.8)		
Comparison site: Pre-intervention IES-R (n = 9)	5 (5.8)		
Post-intervention IES-R (n = 4)	13.1 (20.6)		
Change: Mean (SD)**	6.3 (15.8)		
Pre-intervention GHQ-12 (n = 9)	9.9 (3.4)		
Post-intervention GHQ-12 (n = 4)	14.5 (4.4)		
Change, difference between sites (95% CI)			
IES-R	− 7.3 ( − 31.8, 17.3), p = 0.43		
GHQ-12	− 7.5 ( − 14.4, − 0,7), p = 0.04		

*ITT intention to treat (denominator is all HEMS dispatches in intervention arm) *PP* per protocol (denominator is HEMS dispatches where livestreaming was used).

**Difference between time points not calculated as numbers too small

The stand down rate for HEMS in the intervention arm was lower than expected with only six dispatches out of 20 in the intervention arm occurring in incidents where GoodSAM was used, and only two stand downs due to video livestreaming. Hence, the criteria were met but with very small numbers (Criteria 3). Assessment of psychological harm (Progression Criteria 4) was indeterminate due to very low recruitment of callers and staff within the comparator site. Change over time in IES-R and GHQ-12 scores (from pre- to post- trial, and the difference in change between trial and comparator site) provided no evidence of increased harm after video livestreaming was introduced in staff within the trial site.

### Analysis of study processes

**Fig. 2:**
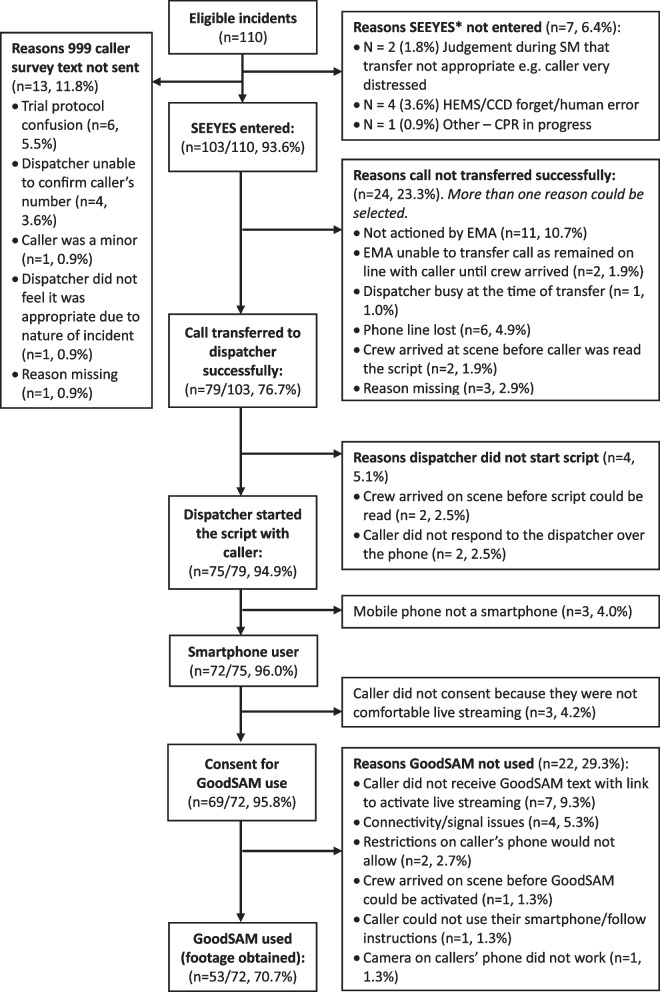
Data flow intervention

Study dataflow for each arm of the study are shown in Figs. 2 (intervention arm) and 3 (control arm). In both arms, in most incidents eligible calls were identified and the code was entered to request transfer to attempt livestreaming (93.6% intervention arm, and 91% control arm). Calls were transferred in 77% of cases (with human or technical errors accounting for those that were not transferred). Only a minority of callers did not have a smartphone (4% of those that could be asked), and consent and successful use of livestreaming was high as per progression criteria 1 and 2 above. Failures were mostly accounted for by technical issues.

**Fig. 3:**
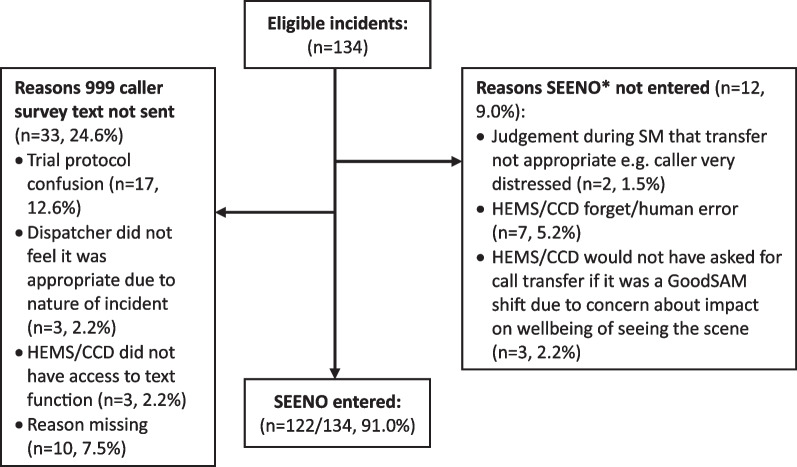
Data Flow Control

### Ancillary analyses (secondary outcomes)

(i) Speed of appropriate dispatch was not significantly different between the control and intervention groups for either HEMS or CCPs (Table 5).
(ii) Stand-down rate (de-escalation). The stand down rate of HEMS was similar in the control and intervention arms (Table 5). In the control arm, 20/53 HEMS dispatches resulted in a stand down (38%). From an ITT perspective, 2/20 HEMS dispatches resulted in stand down in the intervention arm (10%), but only 2/6 dispatches where GoodSAM was used resulted in stand down (33%). Accurate data of stand downs for other SECAmb resources (e.g., Double Crewed Ambulance, DCA) was not feasible to collect in this study due to the constant re-allocation of resources, depending on geography and prioritisation due to urgency.
(iii) Missed calls: only eight calls were identified as ‘missed’ by the HEMS dispatchers/CCD (4 during control shifts and 4 during intervention shifts).
(iv) Request for enhanced resources (HEMS and/or CCPs) from the scene were recorded by the Research Paramedics. Of the 134 incidents in the control arm, there were eight requests (6%) for a HEMS/CCP resource (7 HEMS and 1 CCP) to be sent to the scene, requested by a SECAmb resource already on scene. Of the 110 incidents in the intervention arm, there were three requests (3%) for a HEMS/CCP resource (2 HEMS and 1 CCP) to be sent to the scene. For requests in the intervention arm, one HEMS request and one CCP request had used GoodSAM video livestreaming.

**Table 5:** Speed of (appropriate*) dispatch

Outcome		Control	GoodSAM	Difference	*p*-Value
Speed of dispatch: time from initiation of 999 call to:					
Dispatch of HEMS	N	52	20		
	Time in minutes (95% CI)	19.1 (15.5, 22.7)	17.4 (9.8, 25.0)	1.7 ( − 6.5, − 10.0)	0.67
Dispatch of CCP	N	73	43		
	Time in minutes (95% CI)	9.5 (7.8, 11.1)	8.9 (7.1, 10.3)	0.78 ( − 1.5, 3.1)	0.5
Speed of appropriate* dispatch: time from initiation of 999 call to:					
Dispatch of HEMS*	N	13	6		
	Time in minutes (95% CI)	19.6 (11.0, 28.3)	14.7 (7.1, 22.3)	4.9 ( − 5.5, 15.4)	0.33
Dispatch of CCP*	N	14	8		
	Time in minutes (95% CI)	8.9 (6.5, 11.3)	9.9 (4.6, 15.1)	− 1.0 ( − 6.5, 4.5)	0.69

*For inclusion of the ‘appropriateness’ assessment of dispatch this could only include the sub-sample of patient participants who provided consent for access to medical records. Data are also presented for the whole sample for comparison to this

### Health economic analysis

It was possible to estimate costs of the intervention (£5/ incident) and resources dispatched to incidents (mean (SD) total costs £1403 (2131) control vs £836 (1642) intervention, Additional file 2: Table 2). The proportions of incidents for which deployment of final resources was rated as appropriate were similar between groups (69% control, 71% intervention), although these data were only available for a subsample of incidents due to challenges with patient recruitment. Ambulance resources dispatched per arm and results of cost-consequence analysis are detailed in Additional file 2: Tables 3 and 4.

### Embedded process evaluation

A full list of the research questions for this embedded process evaluation are available elsewhere [18], and comprehensive findings will be published separately. Key findings and exemplar quotes are provided in Additional file 3. For the main trial site, a total of 86 h of observational fieldwork was conducted in the SECAmb EOC, 11 staff interviews (HEMS dispatchers, CCPs and Research Paramedics) and two caller interviews (one who used video livestreaming and one who did not).

In summary, evidence supported that use of GoodSAM video livestreaming was acceptable and easy to use for both callers and EOC staff (with minimal training required). HEMS dispatchers/CCPs found it useful in informing dispatch of EMS resources and qualitative evidence suggested use of video livestreaming in trauma is unlikely to cause any psychological distress to callers in additional to what they may already experience because of witnessing a trauma incident. Similarly, none of the EOC staff were observed to have any visible stressful or emotional reactions to live streamed footage but they did discuss in interviews the potential harm that viewing traumatic scenes could cause if not properly managed. We were able to collect accurate decision-making and dispatch data real time and retrospectively. Application of the criteria to measure appropriateness of dispatch resulted in 97% agreement between Research Paramedics.

In the inner-city sub-study, the Research Fellow completed 200 h of observational fieldwork across 25 shifts (day and night). During these shifts, GoodSAM was used 39 times. Although 34/39 (87%) of the callers who used video livestreaming consented to be sent a survey about their experiences, only seven completed the survey (21% response rate). Of these five agreed to participate in an interview but despite many attempts to arrange interviews, none were completed. As only one caller who completed the survey reported to not speak English fluently and no interviews were possible, the information about diversity of callers using video livestreaming relied predominantly on interviews with staff. Fourteen interviews were conducted with staff (HEMS dispatchers and APP-CCs). Although ethnicity, culture and religious beliefs were not reported to influence decisions to use video livestreaming (and no instances were observed where culture or language presented as a barrier to video livestreaming), some staff reported in interviews that when callers required the use of translation services (e.g., Language Line) this may influence their decision to initiate video livestreaming, especially if the injuries appeared time critical. Furthermore, some HEMS dispatchers/APP-CCs also reported that older age might similarly influence this decision (to request livestreaming) due to their perception that older adults may not have access to and/or find it difficult to use the technology.

## Discussion

The findings from this feasibility RCT can be used to inform the design and conduct of trials in the use of video livestreaming in the pre-hospital setting, and specifically for trauma incidents. We found that video livestreaming was feasible to implement, acceptable and easy to use for both callers and dispatchers, and that it may aid dispatch decision-making. The event rate was as estimated (4.3 eligible incidents per shift, standardised to a 12-h shift) and data regarding decision-making and resource allocation both real-time and retrospectively was feasible to collect. Recruitment of patients involved in incidents and lay public callers remain as major challenges to overcome in future studies.

Given the question remaining over the potential harm that may be caused by use of video livestreaming (to either callers and/or to dispatchers), and in the context of the rapid uptake of such technologies in ambulance services regardless of the sparse evidence regarding this, it is important to prioritise future research that can answer this question. Indeed, one incident reported in the media in another ambulance service suggested that using video livestreaming may have exacerbated the trauma experienced by the caller [35]. In our study whilst we found no direct evidence of harm, interviews suggested that harm may result (or be exacerbated if used for certain types of incidents e.g., suicide attempts. Findings from the inner-city sub-study, which will be reported in full elsewhere, supported the need for governance around if/when/how video livestreaming should be used. Similar conclusions have been reported in the wider literature [14, 35].

Design issues that would need to be addressed for a future study include determining the best timing for randomisation to reduce bias in either arm. For pragmatic reasons, we randomised by working shift for this study, though in the final week found that we could randomise by individual call. We also faced barriers due to governance restrictions and policies within the emergency service that impacted on our ability to recruit callers. Other studies have had similar problems with recruitment of emergency callers [8, 15]. Further work with the lay public and emergency services to agree protocols prior to a future study may help to improve recruitment. Barriers to the use of livestreaming that we report in this study include human error (e.g. failing to transfer calls), but also technology related to the platform and/or callers mobile phones. Such instances were relatively rare but would be important considerations in future studies and for roll-out in practice.

A recurring theme which emerged from the staff interviews was how useful the HEMS dispatchers/CCD found video livestreaming to aid decision making about dispatch. Being able to see the patient and the scene enabled the dispatchers to improve their situational awareness [30], as they were able to gather more information and gain a better understanding of what was happening/had happened at the scene, and could therefore be more confident in their decision-making about the resources required at the scene. Similar findings have been reported elsewhere [3, 8, 14, 36]. The time from initiation of emergency call to dispatch is not a true reflection of time taken for enhanced dispatch to reach the scene, as livestreaming could not commence until the final telephone triage code had been obtained on the NHS Pathways system. This was mandated by the EOC clinical governance processes. Also, an exclusion criterion included emergency resources already being on scene before livestreaming could commence. The average time therefore excludes incidents that were reached quickly.

Research into the impact of technology on dispatch decisions require a method of determining that any impact on decisions was the ‘right’ decision [37]. We have developed and validated a method for determining ‘appropriateness’ of EMS resources sent to incidents within this study, which will be published, in full elsewhere to provide a resource for further studies in this setting.

### Limitations

The low recruitment of patients, callers and EOC staff (from the comparison site) meant that two of the progression criteria were rated as indeterminate. The assessment of appropriateness of resources sent required access to medical records post-incident, and only 44% of patients were approached to request consent for this. There was a very low decline rate (n = 9), with most other patients not being approached due to challenges in identifying or contacting them once they had been discharged from hospital.

### Generalisability

The ambulance trust where the trial took place (SECAmb) is similar in geographical and population size to several other English ambulance trusts and has a similar profile in relation to range of major trauma incidents as other trusts. Future studies of implementation and impact of video livestreaming in ambulance services will need to be designed to consider different dispatch systems and other key differences in operations, including the clinical or non-clinical background of HEMS dispatchers.

### Interpretation and conclusion

The study findings support progression to a future trial, which must be designed to overcome the limitations identified in this feasibility study. Video livestreaming from scene is feasible to implement, acceptable to both callers and dispatchers, and may aid dispatch decision-making, but further assessment of unintended consequences, benefits and harm is required. Given the rapid adoption of such technologies it may be that an alternative research design will be required such as a stepped wedge or realist evaluation, either way embedding a mixed methods process evaluation to robustly understand if/how/why livestreaming works and for whom and in which circumstances.

### Supplementary Information

**Additional file 1**. “Figure S1 and S2 CONSORT”. Title of data: “CONSORT study flow diagram”. This supplementary material includes two CONSORT study flow diagrams for (1) randomisation and eligible incidents and (2) patient recruitment. The file includes information (per condition) about the number of randomised shifts, number of eligible incidents, how many texts were sent to 999 callers, how many surveys were completed by 999 callers and detailed information about the number of patients that were followed-up at hospital and consented.

**Additional file 2**. “Additional File 2: Health Economic Analysis Supplementary Data”. Title of data: “Health Economic Analysis Supplementary Data”. This supplementary material includes four tables: (1) Incident level total healthcare resource use costs (£); (2) Healthcare resource use unit costs; (3) Incident level healthcare resource use (levels of dispatch); and (4) cost-consequences (appropriateness of dispatch at incident level).

**Additional file 3**. “Additional File 3 Process Evaluation Table”.  Title of data: “Summary of key embedded process evaluation questions”. This supplementary material includes a table with all the relevant research questions from the embedded process evaluation, a brief summary of the findings (per question) and example quotes (including the source: surveys, interviews of observations).

## Data Availability

All data requests should be submitted to cath.taylor@surrey.ac.uk for consideration. Data that underlie the results presented here will be shared on reasonable request, once the datasets have been appropriately anonymised including the removal of data from participants who did not consent to their data being shared. The consent process (for patient medical records data, surveys and interviews with callers and staff) included the option to agree (or not) to allow anonymised data to be made publicly available. Not all participants agreed to this.

## Abbreviations

APP-CC: Advanced Paramedic Practitioners in Critical Care
CAD: Computer-aided dispatch system
CAG: NHS Confidentiality Advisory Group
CCD: Critical Care Desk
CCP: Critical Care Paramedic
DCA: Double Crewed Ambulance
EEAST: East of England Ambulance Service NHS Trust
EMA: Emergency Medical Advisor (also known as call handler)
EMS: Emergency Medical Services
EOC: Emergency Operations Centre
GHQ-12: General Health Questionnaire
HEMS: Helicopter Emergency Medical Services
HRA: Health Research Authority
IES-R: Impact of Events Scale Revised
ITT: Intention-to-treat
KSS: Air Ambulance Charity Kent, Surrey, Sussex
LAA: London’s Air Ambulance Charity
LAS: London Ambulance Service NHS Trust
MPDS: Medical Priority Dispatch System
NHS: National Health Service
NIHR: National Institute for Health and Care Research
RAG rating: Red, Amber, Green Rating
RCT: Randomised Controlled Trial
SECAmb: South East Coast Ambulance Service NHS Foundation Trust

## Glossary

APP-CC: Advanced Paramedic Practitioners in Critical Care: A specialist paramedic working with the London Ambulance Service NHS Trust (LAS) with additional post-graduate education, training and experience in prehospital critical care. The role includes working both clinically treating patients and on the Advanced Paramedic Practitioner (APP-CC) desk, dispatching other APP-CCs and providing remote advice to EMS crews
CAD: Computer-Aided-Dispatch system: The software used by Ambulance Trusts to triage calls and dispatch ambulance resources
CCD: Critical Care Desk: the area within the Emergency Operations Centre where the CCPs are dispatched from and from where critical care expertise and support can be sought from the scene of incidents by ambulance crews
CCP: Critical Care Paramedic: A specialist paramedic working with the South East Coast Ambulance Service NHS Foundation Trust (SECAmb) with additional post-graduate education, training and experience in prehospital critical care. The role includes working both clinically treating patients and on the Critical Care Desk (see CCD) dispatching other CCPs and providing remote advice to EMS crews
CPR: Cardiopulmonary resuscitation: Chest compressions given to a person in cardiac arrest
DCA: Double Crewed Ambulance: An emergency ambulance crewed by at least two ambulance service staff who are trained to deliver clinical care at the scene of a medical incident and capable of transporting patients to hospital or another location
Dispatcher: a member of staff within the ambulance service who dispatches appropriate emergency medical resources to the scene of an incident
EMA: Emergency Medical Advisor: a member of staff within the ambulance service who finds out the location of the patient/incident, completes an assessment of the patient, provides live saving instructions (e.g. CPR) and provides reassurance before emergency medical resources reach the patient/scene. Also known as a ‘call taker’ or ‘call handler’
EMS: Emergency Medical Services: Services which provide urgent or emergency medical help
EOC: Emergency Operations Centre: receives and triages 999 calls from members of the public and other emergency services e.g. police, fire and coastguard, and coordinates dispatch of resources to the scene of incidents
GoodSAM Instant-On-Scene: A technology which enables a caller to video livestream from the scene of an emergency to the emergency control centre
HEMS: Helicopter Emergency Medical Services: provide prehospital emergency and critical care to patients via helicopter and/or road, with teams including emergency medical doctors as well as paramedics
KSS: Kent, Surrey, Sussex, used in this study as an abbreviation for Air Ambulance Charity Kent, Surrey, Sussex
Major Trauma Centre: A major trauma centre (MTC) is part of a major trauma network. This type of hospital is dedicated to treating patients with severe injuries resulting from significant trauma. It offers 24/7 emergency services with immediate access to consultants from a broad spectrum of specialised medical services
Major Trauma Network: Coordinated teams and resources dedicated to a specific population, to minimise fatalities and impairments resulting from injuries. These networks are designed to manage the three key stages of a patient's treatment process—pre-hospital, in-hospital, and rehabilitation
MPDS: Medical Priority Dispatch System: A dispatch system which allows the categorisation and prioritisation of emergency medical services, is an alternative to NHS Pathways used in this study
NHS Pathways triage tool: A clinical decision support system supporting the remote assessment of callers to urgent and emergency services, including 999.
Silent Monitoring: When the HEMS dispatchers/CCPs in the EOC silently listen to the conversation between the 999 caller and the EMA to gain more information about the incident
Trauma Unit: A hospital within the major trauma network that caters to all but the most critically injured trauma patients. In situations where reaching a major trauma centre within 45 minutes is not feasible, or if rapid stabilisation is required, patients are initially taken to the closest hospital with a local trauma unit
