# Supplementary material for: The SEE-IT Trial: emergency medical services Streaming Enabled Evaluation In Trauma: a feasibility randomised controlled trial
Source: Scand J Trauma Resusc Emerg Med. 2024 Jan 26;32:7. doi: 10.1186/s13049-024-01179-0 (PMC10883301; doi:10.1186/s13049-024-01179-0)

Additional File 1: Figure S1 and S2 CONSORT

Figure S1 CONSORT study flow diagram: Randomisation and eligible incidents

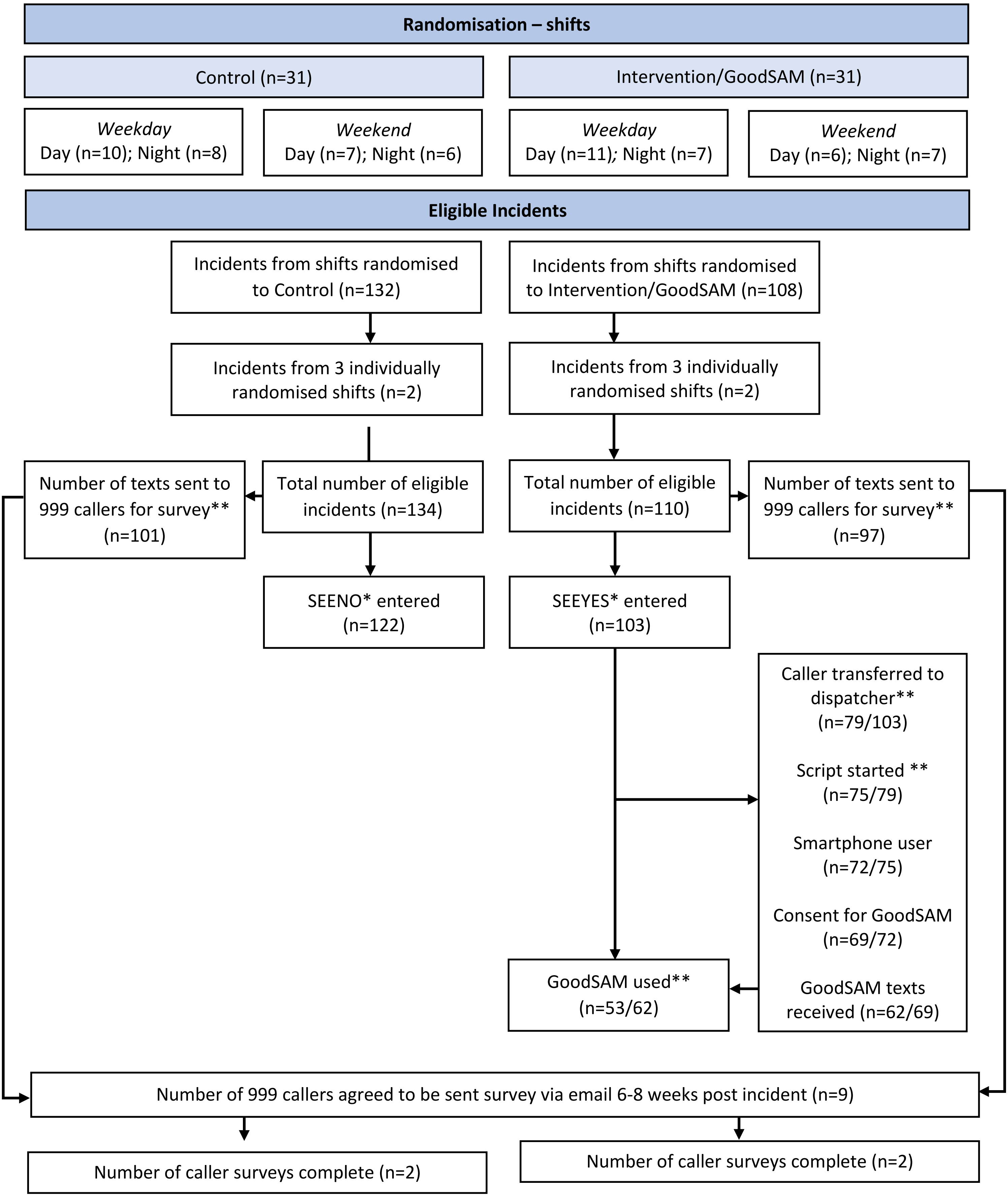

Figure S2: CONSORT study flow diagram: Patient recruitment

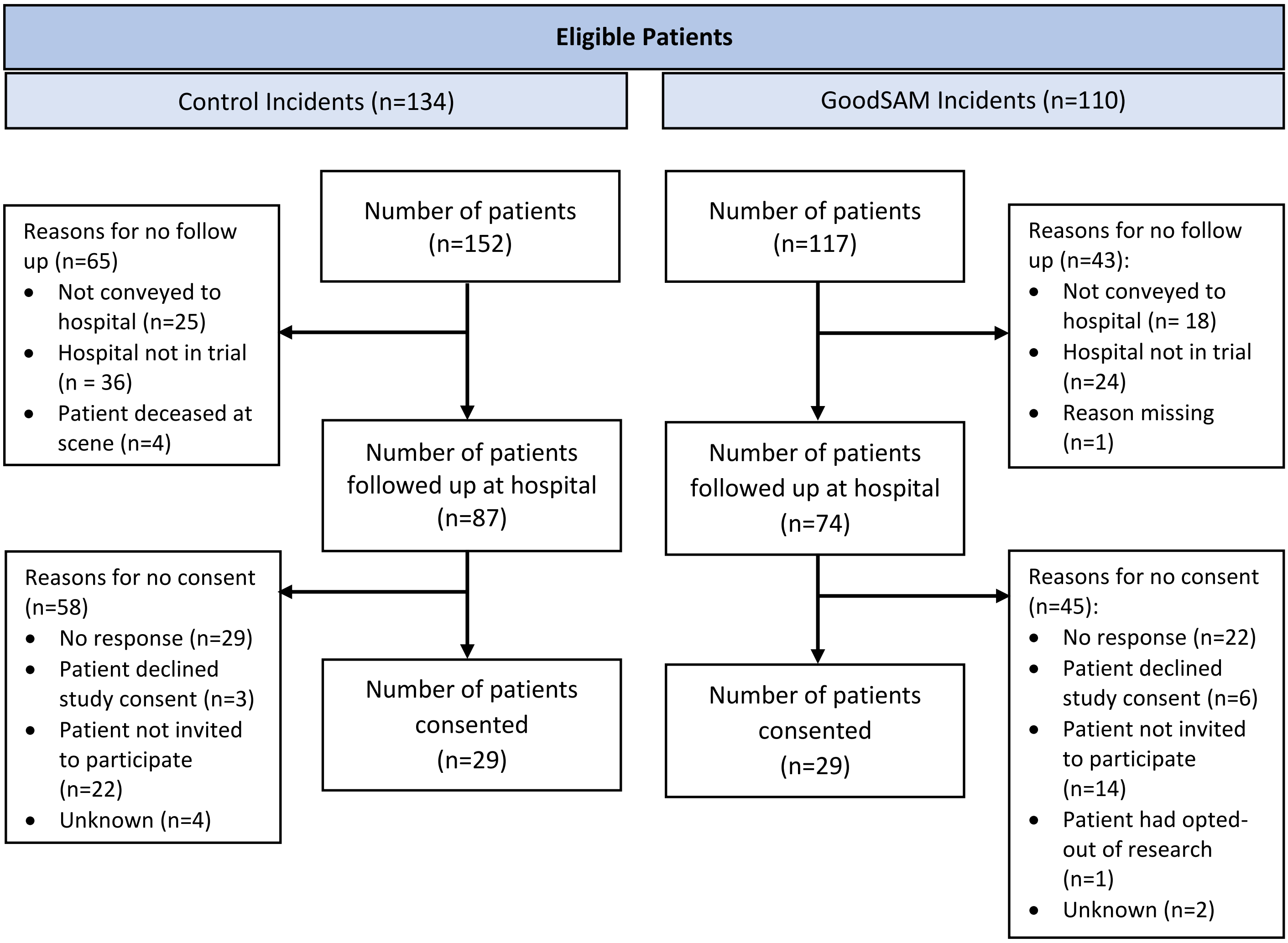

Supplement: Supplementary file 1 — Additional file 1. “Figure S1 and S2 CONSORT”. Title of data: “CONSORT study flow diagram”. This supplementary material includes two CONSORT study flow diagrams for (1) randomisation and eligible incidents and (2) patient recruitment. The file includes information (per condition) about the number of randomised shifts, number of eligible incidents, how many texts were sent to 999 callers, how many surveys were completed by 999 callers and detailed information about the number of patients that were followed-up at hospital and consented. [file 13049_2024_1179_MOESM1_ESM.pdf]
