# Supplementary material for: The SEE-IT Trial: emergency medical services Streaming Enabled Evaluation In Trauma: a feasibility randomised controlled trial
Source: Scand J Trauma Resusc Emerg Med. 2024 Jan 26;32:7. doi: 10.1186/s13049-024-01179-0 (PMC10883301; doi:10.1186/s13049-024-01179-0)
Supplement: Supplementary file 2 — Additional file 2. “Additional File 2: Health Economic Analysis Supplementary Data”. Title of data: “Health Economic Analysis Supplementary Data”. This supplementary material includes four tables: (1) Incident level total healthcare resource use costs (£); (2) Healthcare resource use unit costs; (3) Incident level healthcare resource use (levels of dispatch); and (4) cost-consequences (appropriateness of dispatch at incident level). [file 13049_2024_1179_MOESM2_ESM.pdf]

Additional File 2: Health Economic Analysis Supplementary Data

Unit costs for intervention and ambulance resources used in the economic analysis are shown in the following table.

Table 1: Healthcare resource use unit costs (£)

**Resource use item**	**Unit cost (per minute)**	**Source**	**Notes**
**Intervention costs**<br><br>Video streaming<br><br>HEMS dispatcher<br><br>CCP review	 <br>£0.21<br><br>£0.62<br><br>£1.10	GoodSAM<br><br>PSSRU Unit Costs 2022	£40,000 provision of GoodSAM streaming assuming 100,000 calls.<br>Mean time of call in SEE-IT trial 1.87 minutes.<br><br>Band 4 HEMS dispatcher (£37 per hour) and Band 7 Critical Care Paramedic (£66 per hour)
Road ambulance	£2.68	Ambulance Patient Level Activity and Costing 2019-20<br><br>PSSRU Unit Costs 2022	All submitters: DCA cost £185. Inflated to 21/22 prices.<br>Mean time SECAMB resource mobile in SEE-IT trial (both arms) 73 minutes (min 4, max 269)
CCP	£3.34	Ambulance Patient Level Activity and Costing 2019-20<br><br>PSSRU Unit Costs 2022	All submitters: ECP cost £203. Inflated to 21/22 prices.<br>Mean time CCP resource mobile in SEE-IT trial (both arms) 64 minutes (min 4, max 269)
HEMS Air ambulance	£73.17	Air Ambulance Charity KSS	£4,390 per hour inclusive of overheads
HEMS road ambulance	£17.50	Air Ambulance Charity KSS	£1,050 per hour inclusive of overheads

Mean costs in each arm, with and without the use of GoodSAM video streaming, and separated per type of resource are shown in the following table.

Table 2: Incident level total healthcare resource use costs (£)

**Cost components**	**Control (s.d)**<br>**Incidents = 134**	**Intervention (s.d)**<br>**Incidents = 110**	**Difference**<br>**(95% CI)**
Intervention cost	NA	5 (13)	
Resource item:<br>Road ambulance<br>CCP<br>HEMS	 194 (97)<br>121 (143)<br>1087 (2041)	 181 (86)<br>79 (124)<br>572 (1570)	
Total costs (mean, sd)	1403 (2131)	836 (1642)	-566 (-1055 to 78)

The first resource arriving on scene, the impact of GoodSAM, the total number of responders arriving on scene and the resources conveying patients are shown in the table below.

Table 3: Incident level healthcare resource use (levels of dispatch)

**Resource use item**	**Control** Incidents = 134		**Intervention** Incidents = 110		**Difference**	
	**Number (% of total resources)**	**Per incident**	**Number (% of total resources)**	**Per incident**	**Number (% of total resources)**	**Per incident**
**First resource arriving on scene - number of resources**						
Road	95 (74)	0.71	81 (79)	0.74	-14 (5)	0.03
CCP	32 (25)	0.24	17 (17)	0.15	-15 (-8)	-0.08
HEMS	1 (1)	0.01	1 (1)	0.01	0 (0)	0.00
Total	128	0.96	102	0.90	-26	-0.06
**Change in resources from video - dispatches**						
Road	NA		6 (46)	0.05	NA	
CCP			7 (54)	0.06		
HEMS			0	0.00		
Total			13	0.12		
**Change in resources from video – stand down**						
Road	NA		NA	NA	NA	
CCP			2 (50)	0.02		
HEMS			2 (50)	0.02		
Total			4	0.04		
**Total responder(s) on scene – number of resources**						
Road	206 (66)	1.54	152 (74)	1.38	-54 (9)	-0.16
CCP	72 (23)	0.54	40 (20)	0.36	-32 (-3)	-0.17
HEMS	36 (11)	0.27	13 (6)	0.12	-23 (-5)	-0.15
Total	314	2.34	205	1.86	-109	-0.48
**Resources conveying – number of resources**						
Road	116 (81)	0.87	96 (94)	0.87	-20 (13)	0.01
CCP	18 (13)	0.13	6 (6)	0.05	-12 (-7)	-0.08
HEMS	9 (6)	0.07	0 (0)	0.00	-9 (-6)	-0.07
Total	143	1.07	102	0.93	-41	-0.14
**Total resources dispatched – number of resources**						
Road	206 (62)	1.54	152 (70)	1.38	-54 (8)	-0.16
CCP	74 (22)	0.55	45 (21)	0.41	-29 (-1)	-0.14
HEMS	53 (16)	0.40	20 (9)	0.18	-33 (-7)	-0.21
Total	333	2.49	217	1.97	-116	-0.51

Exploratory cost-consequence analysis based on available appropriateness data is shown in the table below.

Table 4: Cost-consequence (appropriateness of dispatch at incident level)

**Cost/consequence**	**Control (incidents = 29)**	**Intervention (incidents = 28)**	**Difference (95% CI)**
% inappropriate dispatches – over resourced (n)	24 (7)	11 (3)	-13
% inappropriate dispatches – under resourced (n)	7 (2)	18 (5)	11
% appropriate dispatches (n)	69 (20)	71 (20)	2
Mean cost (£) inappropriate dispatches – over resourced (n)	1481 (7)	511 (3)	-970 (-4933 to 2994)
Mean costs (£) inappropriate dispatches – under resourced (n)	591 (2)	225 (5)	-365 (-493 to -238)
Mean costs (£) appropriate dispatches (n)	2940 (20)	1755 (20)	-1185 (-3016 to 646)
Total costs (£)	2426	1348	-1077 (-2492 to 337)
