# Supplementary material for: The SEE-IT Trial: emergency medical services Streaming Enabled Evaluation In Trauma: a feasibility randomised controlled trial
Source: Scand J Trauma Resusc Emerg Med. 2024 Jan 26;32:7. doi: 10.1186/s13049-024-01179-0 (PMC10883301; doi:10.1186/s13049-024-01179-0)
Supplement: Supplementary file 3 — Additional file 3. “Additional File 3 Process Evaluation Table”. Title of data: “Summary of key embedded process evaluation questions”. This supplementary material includes a table with all the relevant research questions from the embedded process evaluation, a brief summary of the findings (per question) and example quotes (including the source: surveys, interviews of observations). [file 13049_2024_1179_MOESM3_ESM.pdf]

Additional File 3: Summary of key embedded process evaluation questions

Research Question	Summary of findings	Example quotes including source
**Is brief software training (<60 mins) feasible to deliver and sufficient?**	Brief training (approx. 20 mins) was provided to most HEMS dispatchers/CCPs, but not all received the training consistently or as intended, which impacted on their confidence to use it. Those that received training felt it was sufficient to use the technology, with confidence growing incrementally after use. Feedback from interviews concurred that use of the technology/GoodSAM was easy and brief training was adequate but should be delivered face-to-face where possible. The software and trial processes were easy to follow but feedback suggested it would have been beneficial to have a trial period of using the technology and processes before the study started. It was clear that confidence and competency increased over time as trial weeks progressed, supporting the benefit of a pre-trial period of use in a subsequent study.	*"I think it needs minimal training, it just needs a couple of practice runs."* HEMS dispatcher interview.<br><br>*"Perhaps something a bit more face-to-face, where they had the option to do it in kind of a sterile environment as opposed to using it… that sort of, hit the ground running, as it were."* HEMS dispatcher interview.<br><br>*"Using GoodSAM is not particularly hard. Some people had issues with like, you know, finding the login, and stuff, page. But that wasn't hard."* CCP interview.
**Is using video acceptable to dispatch control room staff?**	The general consensus from observations, interviews and survey responses was that use of video livestreaming was acceptable, though support and interest varied between and within HEMS dispatchers/CCPs.	*"It's a fantastic concept, like the idea [of GoodSAM] is great"* HEMS dispatcher interview.<br><br>*"I was quite happy to get involved and do bits. Some people just felt that it was one job too many, I guess, and just didn't have the interest in it."* CCP interview.<br><br>*"Both the CCD and the HEMS dispatcher were very keen to use GoodSAM tonight and were disappointed that nothing was coming in for them to have a look at".* RF observation notes.

**Is using video acceptable to 999 callers?**	69/72 (96%) of callers who were asked to activate GoodSAM consented to use the technology (see Additional file 1 for more information). Only three callers did not consent to using video livestreaming, stating they did not feel comfortable to do so. Only two callers were interviewed as part of the study (one that used GoodSAM) but both were overwhelmingly positive about video livestreaming and its acceptability to them.	*"I just found the whole thing…I just thought it was brilliant."* 999 caller interview (used GoodSAM).<br><br>*"The vast majority of people that were asked if we could take hold of their camera were actually quite happy to get involved".* Research Paramedic interview.
**Will/can the public follow instructions?**	62 callers received the SMS text asking them to activate GoodSAM, of whom 58/62 (94%) were able to follow the instructions without any difficulty, and 2/62 (3%) required assistance but then could follow the instructions (see Additional file 1 for more information). Only one caller had partial difficulty (was unable to put their phone onto loudspeaker but was able to transmit images), and one was completely unable to follow the HEMS dispatcher/CCPs instructions. The ease of following the instructions was concurred in interviews and observational fieldnotes.	*"It was easy to understand".* 999 caller interview (used GoodSAM).<br><br>*"It must have been straightforward for me to be able to access it whilst in shock/panic!"* 999 caller survey.
**Is video useful in informing emergency dispatch?**	Qualitative data from observations and interviews supported that video livestreaming was most useful for informing decisions about dispatch by gaining more information about the condition of the patient(s) and visualising what had happened at the scene e.g. impact on vehicles, shape of deformed limbs. The technological features of GoodSAM (audio, visual) were generally reported to be of good quality by the HEMS dispatchers, CCPs and Research Paramedics. The main reported barrier was mobile coverage.	*"It [GoodSAM] really helped in terms of painting a picture of what was going on"* CCP interview.<br><br>*"It certainly gives you a much… generally a much clearer picture, doesn't it, of what's going on? You say see the… the person who's been hit by the car. They say there's a dent in the bonnet, there's a dent in the… in the roof"* Research Paramedic interview.<br><br>*"There's definitely a benefit and we definitely saved some ambulances or sent, you know, specialist resources to patients who needed it sooner than if we hadn't had seen the scene if that makes sense."* HEMS dispatcher interview.

**How is video from multiple calls about the same incident used to inform decision-making?**	There were no attempts to use video livestreaming with more than one caller about the same incident. The consensus from interviews with HEMS dispatchers and CCPs was that where there was more than one caller ringing about the same incident, they generally silent monitored (listened in) each of the calls, and read notes on the CAD, to determine which caller was closest in proximity to the incident and 'sounded' like they would be able to follow instructions to activate video livestreaming (e.g. relatively calm, cooperative, not too distressed or angry).	*"I'd go through, I'd find out who was closest, who is actually able to do it and then, yeah, pick that one."* HEMS dispatcher interview.<br><br>*"We'd still have to listen in to the calls and try and pick the one where the… the caller sounds the calmest."* CCP interview.
**What is the response rate to a follow-up 999 caller survey?**	This was very low (4/244, 2%) and a key area of challenge to be overcome in a future study. Most (198/244, 81%) callers were invited to participate in the study (101/134 callers in the control arm; 97/110 callers in the intervention arm). Only 9/198 callers agreed to participate and be sent a link to complete the survey (to be sent 6-8 weeks later) and of these only 4 completed the survey.	N/A
**Is there any evidence that video livestreaming is associated with risk of psychological harm for 999 callers?**	See also page 17/table 6 for quantitative findings in relation to this. We cannot confidently answer this question due to the very low recruitment rate of callers.<br>Data from caller surveys (N=4), caller interviews (N=2), staff interviews (N=11) and observational data suggests that the using video livestreaming was unlikely to cause additional distress to callers compared to audio only.	*"It [GoodSAM] reassured me that I… you know, we were doing… the people that I and the other people that were there were doing the correct thing, we weren't making the situation any worse."* 999 caller interview (used GoodSAM).<br><br>*"I'm just very relieved it was available on this occasion & I know it will help in the assessment of serious v non-serious/non-urgent situations."* 999 caller survey.
**Is there any evidence that video livestreaming is associated with**	See also page 17/table 7 for the quantitative findings.<br>None of the staff we interviewed reported any negative psychological impact of viewing live streamed images from incidents. This was corroborated by observational data where no visible stressful or emotional reactions were observed except	*"There was nothing that really concerned me that I saw."* CCP interview.

**risk of psychological harm for staff who view the streamed footage?**	for one incident where the HEMS dispatcher said they did not want to livestream (leading to a protocol change to enable and capture this). No further incidents occurred in the study. However, staff did feel that there was potential for harm to be caused by viewing traumatic scenes. They also mentioned that there were certain incidents that they would be hesitant to use video livestreaming as it may cause additional distress for them (e.g. violent suicide attempts, and patients with injuries incompatible with life).<br>Although no CCPs reported any harm associated with using GoodSAM, some did report 'frustration' when the EMA did not transfer the call through, the caller did not follow their instructions and when the technology did not work properly. Some staff felt that having a clinical background made a difference to risk, and others disagreed with this.	*"They were all very mild. I can't really think of anything that we saw and went, 'Woah' or anything that made us particularly uncomfortable."* Research Paramedic interview.<br><br>*"The footage itself, none of it kind of bothered me at all".* HEMS dispatcher interview.
**Can we collect decision data real time and obtain accurate follow-up decision data retrospectively?**	Yes – the 'real time' proforma completed by the Research Paramedics (observing all trial shifts) was reported to be easy to use. Data entries were checked for accuracy and few errors had to be corrected. Dispatch decisions could be easily checked for accuracy retrospectively. Changes to dispatch of DCAs was not easy to collect due to multiple changes that occur as they are re-routed for clinical priority or geographical reasons.	N/A
**Can appropriateness by reliably measured?**	The algorithms developed by the expert panel were applied by two Research Paramedics independently and resulted in 97% agreement. Those that were not agreed, and all that resulted in under or over resourced judgements were also reviewed by the expert panel, leading to some further amendments to the criteria. The criteria and methods for developing and validating them will be published in full elsewhere.	N/A
**What is the potential for contamination?**	No contamination was reported in this trial between control and intervention arms. It is possible that control group incidents may be incorrectly allocated (and use video livestreaming) if	N/A

	the wrong codes are entered. This may be more likely if randomisation is by call rather than shift, but we did not find evidence of this.	
**How does the total call length compare between intervention and control arms?**	Due to the use of NHS Pathways (22) the intervention arm calls had to be an extension to the standard NHS Pathways call as 999 callers could not be transferred until that process had ended. However, GoodSAM systems data confirmed the average video livestreaming time was 6.07 minutes (95% CI [3.31, 8.83]).	N/A
